# Retained primary teeth in STAT3 hyper-IgE syndrome: early intervention in childhood is essential

**DOI:** 10.1186/s13023-020-01516-3

**Published:** 2020-09-10

**Authors:** Iris Meixner, Beate Hagl, Carolin I. Kröner, Benedikt D. Spielberger, Ekaterini Paschos, Gregor Dückers, Tim Niehues, Ronny Hesse, Ellen D. Renner

**Affiliations:** 1 University Children’s Hospital, Dr. von Haunersches Kinderspital, Ludwig Maximilian University, Munich, Germany; 2 Oral and maxillofacial surgery, Ludwig Maximilian University, Munich, Germany; 3 Chair and Institute of Environmental Medicine, UNIKA-T, Technical University of Munich and HelmholtzZentrum München, Munich/Augsburg, Germany; 4 Department of orthodontics, Ludwig Maximilian University, Munich, Germany; 5 HELIOS Children’s Hospital, Krefeld, Germany

**Keywords:** Retained primary teeth, Delayed eruption of permanent teeth, Root resorption, Hyper-IgE syndrome, HIES, STAT3, STAT3-HIES, Primary immunodeficiency, PID

## Abstract

**Background:**

STAT3 hyper-IgE syndrome (STAT3-HIES) is a rare primary immunodeficiency that clinically overlaps with atopic dermatitis. In addition to eczema, elevated serum-IgE, and recurrent infections, STAT3-HIES patients suffer from characteristic facies, midline defects, and retained primary teeth. To optimize dental management we assessed the development of dentition and the long-term outcomes of dental treatment in 13 molecularly defined STAT3-HIES patients using questionnaires, radiographs, and dental investigations.

**Results:**

Primary tooth eruption was unremarkable in all STAT3-HIES patients evaluated. Primary tooth exfoliation and permanent tooth eruption was delayed in 83% of patients due to unresorbed tooth roots. A complex orthodontic treatment was needed for one patient receiving delayed extraction of primary molars and canines. Permanent teeth erupted spontaneously in all patients receiving primary teeth extraction of retained primary teeth during average physiologic exfoliation time.

**Conclusions:**

The association of STAT3-HIES with retained primary teeth is important knowledge for dentists and physicians as timely extraction of retained primary teeth prevents dental complications. To enable spontaneous eruption of permanent teeth in children with STAT3-HIES, we recommend extracting retained primary incisors when the patient is not older than 9 years of age and retained primary canines and molars when the patient is not older than 13 years of age, after having confirmed the presence of the permanent successor teeth by radiograph.

## Background

STAT3 hyper-IgE syndrome is a rare primary immunodeficiency associated with eczema, recurrent infections, and high serum IgE levels [1, 2]. Due to the clinical overlap with atopic dermatitis, diagnosis is often delayed until irreversible complications, such as chronic lung disease, already have occurred [3]. Early diagnosis has been improved by identifying heterozygous dominant-negative mutations in the signal transducer and activator of transcription 3 (*STAT3*) gene in 2007 and corresponding molecular testing [4–6]. The impaired STAT3 signaling in STAT3-HIES leads to defective B cell development and antibody maturation [7–9] as well as reduced Th17 cell numbers [10, 11]. As a consequence, patients often suffer from mucocutaneous candidiasis, recurrent skin and lung infections with *Staphylococcus aureus,* and respiratory tract infections, particularly with *Haemophilus influenzae* and *Streptococcus pneumoniae* [1, 2]. To prevent infections and the associated complications, treatment includes long-term antibiotic treatment with co-trimoxazole or a *Staphylococcus aureus* targeted cephalosporin, immunoglobulin substitution therapy, and as needed antifungal treatment [3, 8, 12].

Moreover, non-immunologic findings such as scoliosis, pathologic fractures, characteristic facies, a high arched palate, midline defects, and retained primary teeth have been reported in STAT3-HIES [1, 2, 13–16]. Thus, not only do infection associated complications impact patients’ quality of life, but the retention of primary teeth might also cause significant impairments. Most persistent primary teeth remain functional for many years; however, persistence might lead to clinical problems, including profound caries, periodontitis, and infra-occlusion [17]. Abnormal shedding of primary teeth may also occur in otherwise healthy individuals. The most common reason of persistence of primary teeth is the congenital absence of the permanent successor tooth [17–19]. Ankylosis of primary teeth and impaction, abnormal position, and late eruption of successor teeth may likewise cause retention of permanent teeth [19].

In this paper, we assess the dental history of 13 patients with STAT3-HIES from childhood into adulthood focusing on the exfoliation of primary teeth to raise awareness of STAT3-HIES and dental treatment in STAT3-HIES patients to help prevent dental complications.

## Results

All patients carried a heterozygous *STAT3* mutation and fulfilled the characteristic findings of STAT3-HIES consisting of eczema, elevated serum-IgE, recurrent infections, and associated skeletal findings (Supplementary Table 1). The median age was 20 years of age (range 5 to 48 years of age) with an almost equal gender distribution (six female, seven male). All patients received antibiotic prophylaxis, immunoglobulin substitution therapy and were on antifungal treatment and regular inhalation therapy if needed.

### Dental history of two STAT3-HIES patients

The dental histories of two exemplary patients show the outcome of different treatment strategies: In patient #4 the primary teeth were all extracted at the average exfoliation time, while patient #3 received some tooth extractions later in life.

**Fig. 1:**
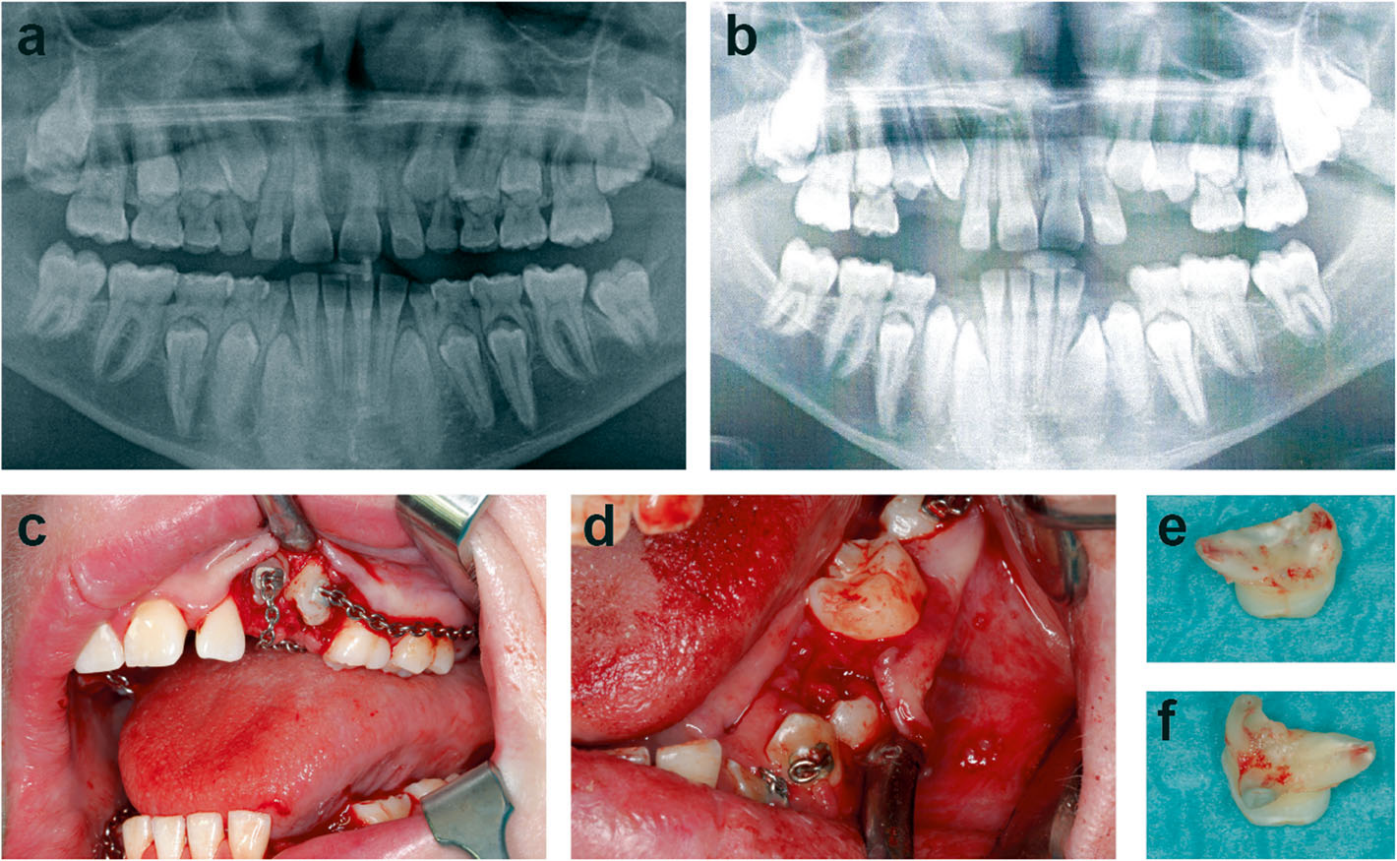
Complex orthodontic treatment to induce permanent teeth eruption in a STAT3-HIES patient after delayed primary teeth extraction. Made when patient #3 was 15 years of age, the panoramic radiograph shows 4 persistent primary canines and all 8 primary molars. Maxillary second permanent molars have not yet reached the occlusal level (**a**). One year after extraction of the primary predecessors the permanent canines and first and second premolars of patient #3 were still impacted (**b**). Intra-operative pictures of the extraction of the primary first and second molars with fixation of the orthodontic treatment on the permanent teeth and subsequent bone ablation at the age of 17 are shown (**c**, **d**). The extracted second primary molars of patient #3 at 17 years of age have almost no root resorbtions (**e**, **f**)

Primary teeth eruption was unremarkable in patient #3 during the average eruption time. The primary incisors of patient #3 were extracted between 7 and 9 years of age, with the exception of 2 incisors which exfoliated spontaneously, and the permanent incisors then erupted shortly afterwards. The primary canines and molars did not exfoliate spontaneously and persisted during the physiologic eruption period of the corresponding successor teeth. When patient #3 was 15 years of age, all 4 retained canines were extracted; however, the successor teeth remained impacted within the jaw. In order to create additional space for the permanent canines, the primary first molars were removed at 16 years of age. The successor teeth still failed to erupt (Fig. 1a, b). Therefore, bone ablation superior of the impacted permanent teeth was performed under anaesthesia followed by a complex orthodontic treatment to pull the permanent canines and first premolars into the oral cavity. After this operation, the canines and first premolars began to erupt. At 17 years of age, the last 4 primary teeth were removed and the second premolars were included in the orthodontic treatment (Fig. 1c, d). Finally, the top of the second premolars erupted 3 weeks after the final operation and all permanent teeth, which had been included in the orthodontic treatment, had erupted. The extracted primary molars showed almost completely unresorbed roots (Fig. 1e, f). To align the dental arch and to ensure optimum tooth positioning, over two years of complex orthodontic treatment followed.

**Fig. 2:**
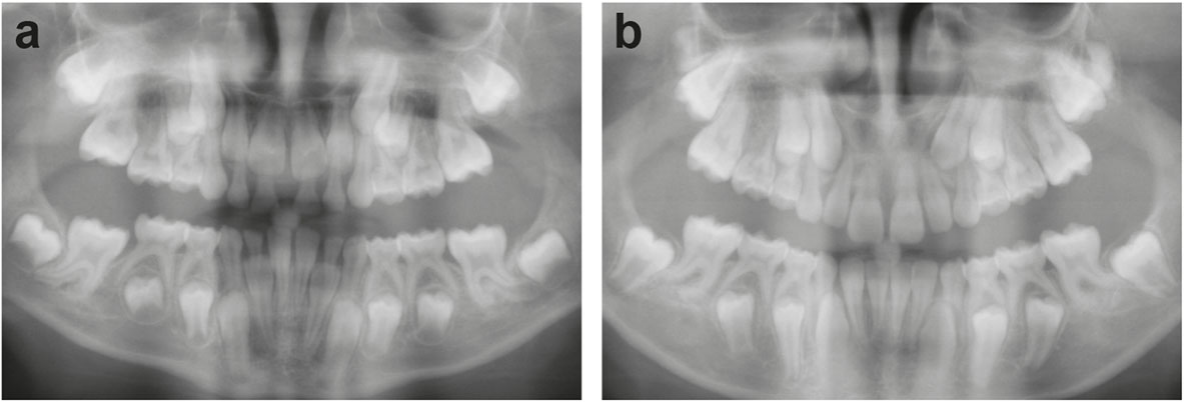
Permanent teeth eruption after timely extraction in a STAT3-HIES patient. At 8 years of age, patient #4 had 18 retained primary teeth, whose roots show very little root resorption in a panoramic radiograph (**a**). Subsequently, mandible and maxillary central and lateral primary incisors were extracted and the panoramic radiograph at 12 years of age (**b**) shows that all successor teeth erupted spontaneously. Agenesis of maxillary second premolars is shown. The canines were extracted at 13 years of age and erupted shortly afterwards (not shown)

In patient #4 all primary mandible and maxillary central and lateral incisors were extracted prior to the age of 9. The permanent successor teeth erupted shortly afterwards (Fig. 2a, b). A radiograph at 12 years of age showed unresorbed and only slightly resorbed roots for the remaining primary teeth. The patient’s remaining primary teeth were all subsequently extracted before the age of 13 with the permanent successor teeth erupting shortly afterwards. Due to timely teeth extraction, all primary teeth erupted without problem within the average eruption time.

### Primary teeth: eruption, spontaneous exfoliation, retention, and root resorption in STAT3-HIES

**Fig. 3:**
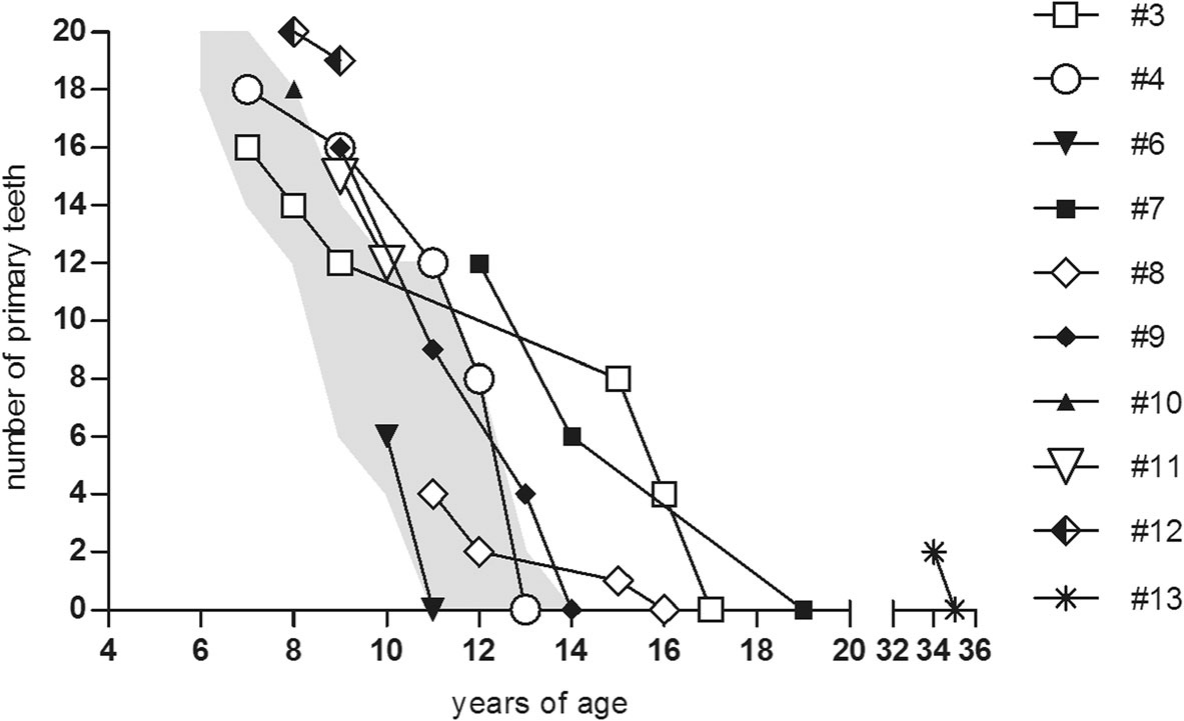
Course of spontaneous exfoliation and primary tooth extraction in STAT3-HIES patients. The number of primary teeth remaining in the oral cavity is shown in all patients except for patient #2, who had not yet reached the age of teeth shedding, and patient #1 and #5 with inconspicuous shedding of primary teeth. The grey area represents the average exfoliation period of primary teeth according to Logan and Kronfeld [20]. Due to timely extraction, the primary teeth exfoliation of patients #6 and #10 is within the average age range

Eruption time, tooth size, tooth appearance, and the number of primary teeth were unremarkable in all 13 STAT3-HIES patients. Due to age, patient #2 (5 years of age) had not yet lost any primary teeth. In 2 patients (#1, #5) all primary teeth exfoliated naturally within the normal age range, while 10 patients (83%) experienced retained primary teeth (2 to 20 teeth per patient) and delayed eruption of permanent teeth up to 35 years of age (Fig. 3, Table 1). In 3 patients (#4, #6, #9) no primary tooth exfoliated spontaneously within the normal age range. Two patients (#10, #12) had retained incisors, while the presence of their primary canines and molars was appropriate to age. In 5 patients (#3, #7, #8, #11, #13), both spontaneous exfoliation and retention of 2 to 8 incisors, up to 2 canines, and up to 6 molars was observed. Thus, only 36% of incisors, 29% of canines, and 27% of molars exfoliated spontaneously within the physiological exfoliation time [20] in the STAT3-HIES patients with retained primary teeth. In all these patients, except for patients #3 and #12, the first and second permanent molars erupted on time (first molar: 5.5–7 years of age, second molar: 12–14 years of age). The first permanent molars of patient #12 appeared with a delay of approximately 2 years at 9 years of age, and the maxillary second permanent molars of patient #3 had not yet reached the occlusal level at 17 years of age.

**Table 1:** Dental and oral findings of STAT3-HIES patients

ID	Age [years]	Gender	retained primary teeth	extracted primary teeth (No.)	delayed resorption of primary teeth (as to X-ray)	oral candidiasis	gingivitis	aphthous ulcers	abnormal dental anatomy	other findings	Heterozygous STAT3 mutation
#1	37	female	no	no	no	yes	no	no	no	no	c.1144C > T; p.R382W
#2	5	male	N/A	N/A	no X-ray available	no	no	no	no	median rhomboid glossitis	c.1144C > T; p.R382W
#3	17	male	yes	yes (18)	yes	yes	no	yes	no	no	c.1145G > A; p.R382Q
#4	12	female	yes	yes (20)	yes	yes	no	no	yes	agenesis of 2 premolars	c.1909G > A; p.V637M
#5	32	male	no	no	no	no	yes	no	no	no	c.1152 T > A; p.F348L
#6	16	female	yes	yes (20)	yes	no	no	no	no	no	c.1145G > A; p.R382Q
#7	19	female	yes	yes (7)	yes	no	no	no	yes	abnormal shaped premolar	c.2114 A > C; p.Y705C
#8	23	female	yes	yes [4]	yes	no	no	yes	no	no	c.1406A > G; p.Q469R
#9	24	female	yes	yes (20)	yes	yes	yes	no	no	no	c.1145G > A; p.R382Q
#10	7	male	yes	yes (2)	no X-ray available	no	yes	no	no	no	c.1145G > A; p.R382Q
#11	9	male	yes	yes (5)	yes	yes	yes	no	no	no	c.1145G > A; p.R382Q
#12	8	male	yes	yes (1)	yes	yes	no	no	yes	agenesis of 1 premolar	c.1144C > T; p.R382W
#13	48	male	yes	no	no X-ray available	yes	no	no	no	no	c.1144C > T; p.R382W
Positive for finding:			83%	75%	80%	54%	31%	15%	15%	8%	

N/A: not applicable

Delayed primary tooth root resorption was observed in 8 of the 10 (80%) STAT3-HIES patients older than 8 years of age, for whom radiographs were available. Detailed root resorption was assessed in 22 panoramic radiographs (Supplementary Table 2) in 3 age groups: (a) up to 9 years of age when physiologically all incisors should have exfoliated, (b) 10 to 13 years of age when canines and molars also exfoliate, and (c) older than 13 years of age when no primary teeth should remain.

**Fig. 4:**
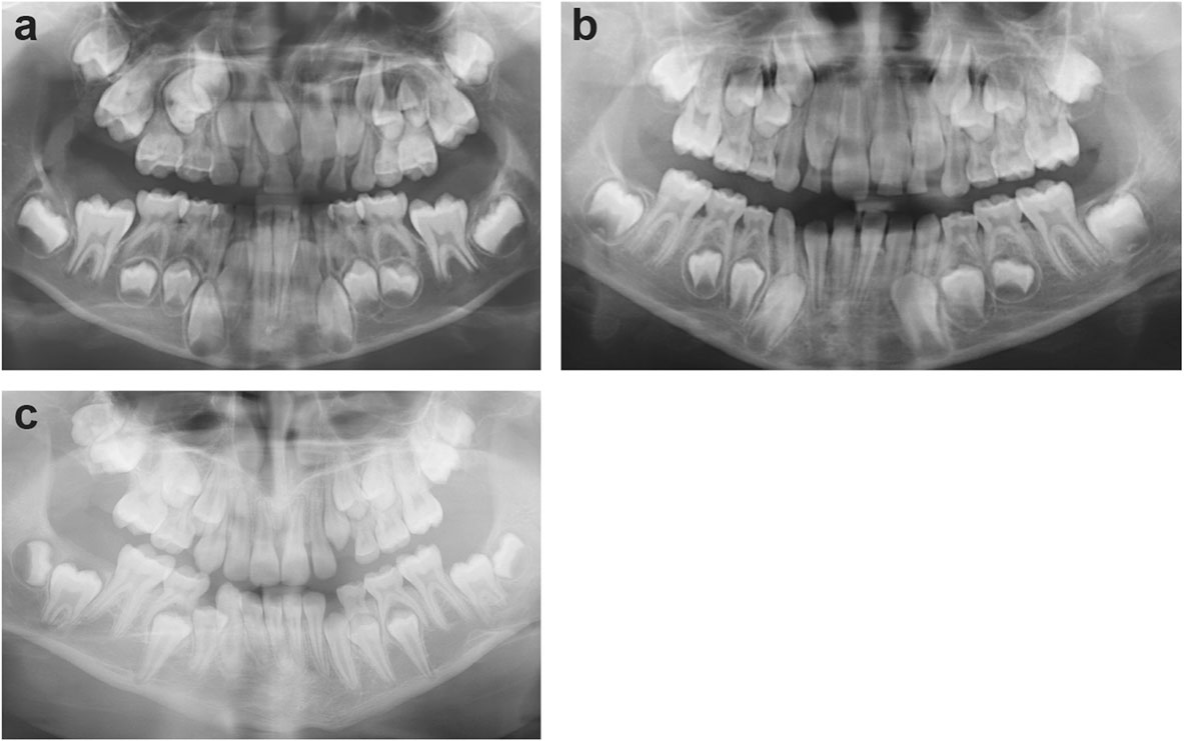
Panoramic radiographs of STAT3-HIES patients with delayed root resportion. (**a**) The panoramic radiograph of patient #12 shows persistence of all primary teeth with delayed root resorbtion of most primary teeth at 8 years of age. The first permanent molars have not yet reached the occlusal level while the superior alveolar bone had already been resorbed. (**b**) At 9 years of age, patient #11 had 3 retained primary incisors, primary canines and molars with unresorbed and barely resorbed primary tooth roots. (**c**) At 10 years of age patient #6 had multiple retained primary molars in the maxilla and mandible with unresorbed and barely resorbed tooth roots with completed bone resorption superior to the second permanent molars on the left side and almost complete resorption on the right side

Between 8 and 9 years of age, 4 patients (#4, #9, #11, #12) showed delayed root resorption of primary teeth with unresorbed roots in incisors, canines, and molars (Fig. 4). Between 10 and 13 years of age, 4 patients (#4, #6, #7, #9) still had up to 4 primary canines and 4 to 8 primary molars, of which some had unresorbed roots. The assessment of panoramic radiographs in 3 patients (#3, #7, #8) above 13 years of age revealed unresorbed primary tooth roots in 3 primary canines in patient #3 and in one primary molar in patient #7.

### Extraction therapy of retained primary teeth, orthodontic and conservative treatment

Nine patients (#3, #4, #6 to #12) had from 1 to 20 primary teeth per patient extracted between 7 and 17 years of age to enable eruption of permanent teeth. In 7 of the 9 patients sequential tooth extraction was required, leading to series of 2 to 8 extractions per patient. In 8 of 9 STAT3-HIES patients, permanent teeth erupted shortly after the primary teeth had been extracted around the physiological exfoliation age.

Nine patients received an orthodontic treatment including removable or fixed dental braces. In patient #9 one permanent premolar in each quadrant had to be removed for orthodontic reasons to create more space for the remaining teeth. To correct the nasal and upper jaw configuration, patient #1 received an osteotomy of the maxilla including orthodontic correction. Five patients (#1, #5, #7, #8, #13) needed conservative dentistry, while the other 8 patients had no fillings, endodontic treatments, or inlays.

### No complications after extraction or orthodontic treatment

Despite repeated oral candidiasis in 7 (54%), recurrent aphthous ulcers (1–12 times per year) in 2 (15%), and gingivitis in 4 (31%) patients, there were no infections after tooth extraction or orthodontic treatment in any of the patients (Table 1). Wound healing was unremarkable in all patients after extractions. As a precaution, patient #3 was given amoxicillin/clavulanic acid (875/125 mg, twice/day) from the day of complex dental treatment until fifth postoperative day, in addition to the patient’s standard prophylactic co-trimoxazole antibiotic treatment 3 times a week and immunoglobulin substitution therapy.

### Abnormal dental and oral anatomy

**Fig. 5:**
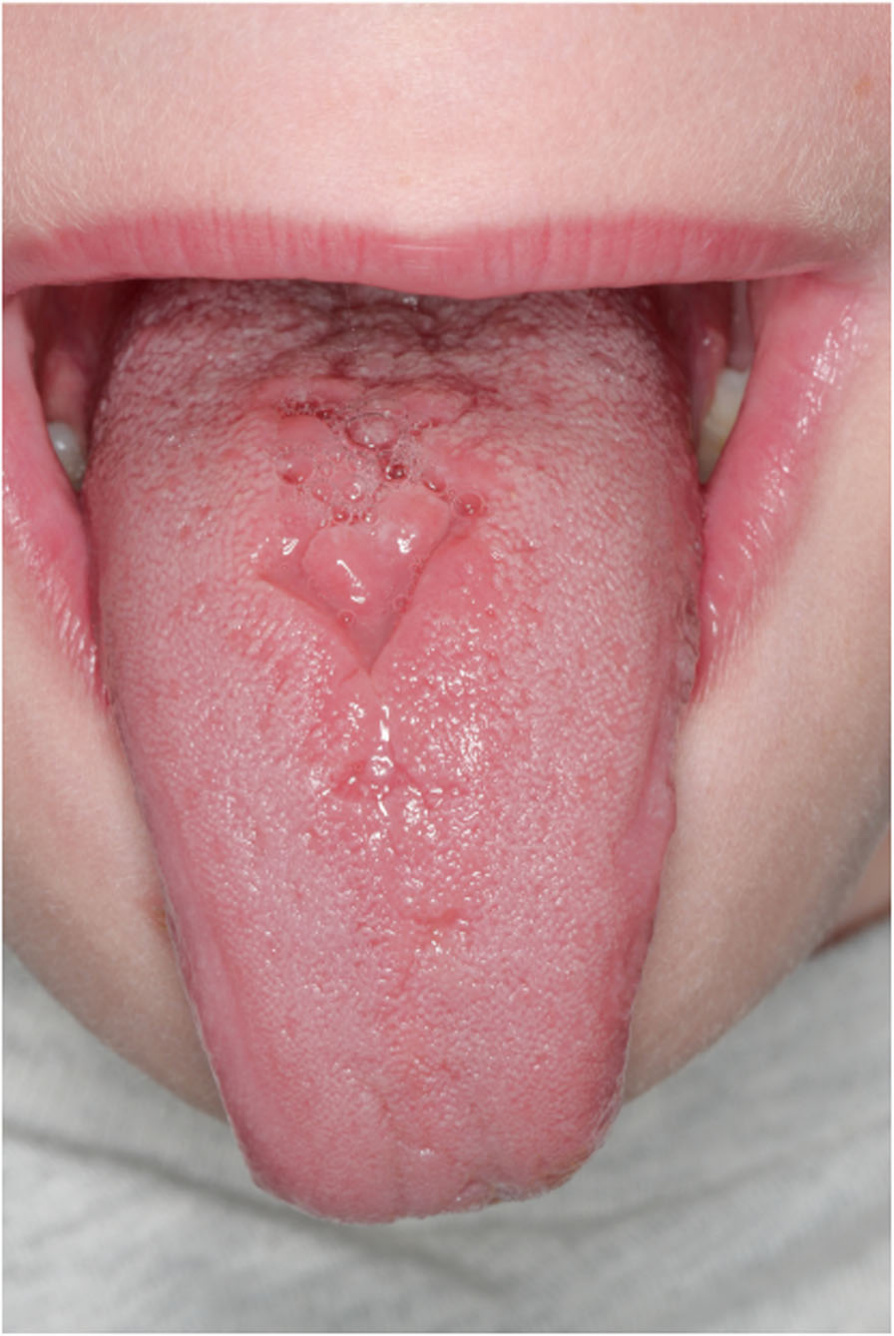
Midline defect in a STAT3-HIES patient. Abnormal fissuring of the tongue in patient #2 consisting of an asymptomatic 2 mm deep midline v-shaped and a pyramidal cleft located anterior of the circumvallate papillae

Three patients (#4, #7, #12) had abnormal dental anatomy in permanent teeth: agenesis of both maxillary second premolars in patient #4, an agenesis of the right-sided maxillary second premolar in patient #12, and an abnormal sized right-sided mandibular second premolar in patient #7. In 4 patients (#3, #8, #10, #11) permanent incisors were temporarily present lingual together with the primary incisors until the primary teeth were extracted. Patient #2 had an abnormal fissuring of the tongue (Fig. 5).

## Discussion

In this report, we focused on dental and oral findings in STAT3-HIES patients to raise awareness of an optimized dental treatment to prevent dental complications in adolescence.

In addition to retaining primary teeth, STAT3-HIES patients have various oral manifestations, such as high arched palates and midline defects [1, 13–16]. Accordingly, one of our patients had a mild abnormal fissuring of the tongue, which is also reported in other STAT3-HIES patients and may consist of large tongue lesions with deep midline clefts anterior to the circumvallate papillae [15]. Gingivitis (31%) and aphthous ulcers (15%) were frequent findings in our STAT3-HIES cohort, however comparable to frequencies observed in the general population.

While primary tooth eruption was unremarkable, 83% of our patients showed persistent primary teeth with delayed primary tooth exfoliation and permanent tooth eruption; thus, persistent primary teeth were found slightly more frequently compared to 64 to 75% of STAT3-HIES patients in previous reports [1, 13, 14]. One patient had a premolar almost twice the normal size. The congenital absence of the permanent successor tooth as the most common cause of primary tooth retention [18, 19] was only observed on one tooth each in 2 patients.

Tooth eruption is a physiologic process, which is still not completely understood. Various tissue changes, such as resorption and apposition of the alveolar bone, and resorption of the primary tooth root have been associated with it [21, 22]. Whereas bone undergoes constant physiological turnover, the resorption of primary tooth roots only occurs during the change from primary to second dentition [23].

On a cellular level, osteoclasts and odontoclasts have been linked to tooth eruption, and their dysregulation has been correlated to abnormal shedding of primary teeth [24]. In STAT3-HIES patients, the increased number of osteoclasts has been suggested to cause skeletal symptoms such as scoliosis and pathologic fractures [25]. As osteoclasts are essential to resorb the alveolar bone to form an eruption pathway [26, 27], the increased number of osteoclasts might explain the unremarkable formation of the eruption pathway in the alveolar bone. Consequently, primary teeth and permanent teeth after timely primary tooth extraction erupted without problems in STAT3-HIES patients.

Delayed resorption of primary tooth roots has been hypothesized to cause persistence of primary teeth in STAT3-HIES [1, 13, 16]. This hypothesis is supported by our finding of a more frequent spontaneous exfoliation of the incisors which have only one smaller root. In contrast, canines, whose roots reach deeper, and molars, which are fixed with two or three roots, were more likely to persist. Impaired root resorption in STAT3-HIES has been suggested to result from abnormal persistence of Hertwig’s epithelial root sheath (HERS) on the surface of extracted primary teeth [13]. However, the tooth roots of mice with normal dentition are also reported to show persistence of some HERS cells that are incorporated by the thickening cementum layer [28]. Thus, the persistence of HERS cells in STAT3-HIES may also be a physiologic phenomenon. Another contributing factor to limited root resorption might be the impaired function of odontoclasts. Both osteoclasts as well as odontoclasts are thought to be regulated by the extracellular matrix protein osteopontin (OPN), whereas an OPN-deficiency leads to a more substantial decrease in odontoclastic than osteoclastic activity [24, 29, 30]. Reduced OPN expression has been reported in STAT3-HIES patients and has been theorized to result in diminished activation of odontoclasts and consequently in the retention of primary teeth [31]. Functional yet slower primary tooth root resorption likely explains why in some STAT3-HIES patients (also in our cohort), particularly primary teeth with only one root can exfoliate naturally without intervention in adolescence or even adulthood.

Our assessment, and particularly the complex orthodontic treatment of patient #3, however, underlines the necessity of extracting primary teeth in STAT3-HIES patients during the physiological exfoliation range of the primary tooth. If primary teeth were extracted later in life, we observed impaction of permanent successor teeth. In a previous report orthodontic extrusion of impacted teeth in STAT3-HIES patients was not recommended due to increased enamel fragility and carioreceptivity [16]. Yet even in patient #3, with multiple extractions, bone ablation, and subsequent complex orthodontic extrusion of impacted permanent teeth, none of the anticipated complications were observed. None of our patients had complications such as wound healing issues or infections of the extraction wounds. As precaution, however, we recommend antibiotic prophylaxis, such as amoxicillin/clavulanic acid, prior to complex dental treatment, in addition to the continuous medication STAT3-HIES patients receive.

## Conclusions

In conclusion, permanent teeth erupt normally in STAT3-HIES patients if retained primary teeth are extracted around the physiological exfoliation age. Complications such as impaired wound healing after extraction were not observed. However, to limit infection risks antibiotic treatment, such as amoxicillin/clavulanic acid, was started on the day of complex dental treatment in addition to continuing the patient’s standard antibiotic and immunoglobulin substitution therapy. Prior to tooth extraction, we advise confirming the presence of the successor tooth by radiograph. The awareness of the association of STAT3-HIES with retained primary teeth is important for primary care physicians as well as for dentists, who can then optimize dental treatment by timely tooth extractions and avoid complications, such as impaction of permanent teeth within the jaw.

## Methods

To optimize dental management, we evaluated the development of the dentition and long-term outcomes of dental treatment in STAT3-HIES patients. Sixteen patients followed at the University Children’s Hospital of the Ludwig Maximilian University (LMU) Munich were asked to complete a dental history questionnaire. Thirteen STAT3-HIES patients (6 females and 7 males, median 20 years of age, range: 5 to 48 years of age) from 11 unrelated families agreed to take part in the study (Supplementary Table 1). The patients’ families and dental care physicians were asked to assist in completing the questionnaire. Inclusion criteria for participation were characteristic clinical findings of STAT3-HIES and a confirmed disease-causing heterozygous *STAT3* mutation. Exclusion criteria were dental abnormalities without any additional findings of STAT3-HIES and the lack of a genetic STAT3 defect.

All patients except for patient #12 have been previously described [2, 11, 32, 33]. Their STAT3-HIES diagnoses have been molecularly defined with diagnostic testing, such as reduced Th17 cell counts and Sanger sequencing of the *STAT3* gene as previously described [11]. Primer sequences are available upon request. Mutations were reported using the nomenclature of den Dunnen and Antonarakis [34].

Thirty-one radiographs of 11 patients were available, including radiographs provided by the local dental care physicians. Radiographs were not uniform since performed during regular dental care appointments by different dental care physicians. Twenty-two panoramic radiographs (PANs) of 9 patients (3 analogue / 19 digital) and 9 periapical radiographs of 3 patients (4 analogue/ 5 digital) were included. PANs were assessed focusing on number of primary teeth, number of retained permanent teeth, agenesis, conservative dentistry, orthodontic treatment, and status of root resorption of primary teeth (no resorption, resorption) (Supplementary Table 2). Periapical radiographs were assessed concerning periapical status of the teeth and conservative dentistry (Supplementary Table 3). The Universal Numbering System was used. Physiological age ranges of primary tooth exfoliation and permanent tooth eruption were defined according to the standards of the American Academy of Pediatric Dentistry defined according to Logan and Kronfeld [20].

Written informed consent was obtained and the study was approved by the LMU review board (#381–13). Figures were generated with GraphPadPrism 5.0 (GraphPad Software Inc., San Diego, CA, USA) and Adobe Illustrator CS6 (Adobe Systems Inc., San José, CA, USA).

## Supplementary information

**Additional file 1:** **Supplementary Table 1.** Clinical and molecular findings of the STAT3-HIES patients.

**Additional file 2:** **Supplementary Table 2.** Dental findings in panoramic radiographs.

**Additional file 3:** **Supplementary Table 3.** Dental findings in periapical radiographs.

## Data Availability

All data generated or analysed during this study are included in an anonymized form in this article and its supplementary information files except for the individual radiographs and the questionnaires. The entire set of radiographs analysed during this study and a copy of the blank questionnaire are available from the corresponding author upon reasonable request. To protect the patients’ privacy, completed questionnaires are not publicly available.
