# Supplementary material for: Retained primary teeth in STAT3 hyper-IgE syndrome: early intervention in childhood is essential
Source: Orphanet J Rare Dis. 2020 Sep 10;15:244. doi: 10.1186/s13023-020-01516-3 (PMC7488068; doi:10.1186/s13023-020-01516-3)
Supplement: Supplementary file 1 — Additional file 1: Supplementary Table 1. Clinical and molecular findings of the STAT3-HIES patients. [file 13023_2020_1516_MOESM1_ESM.pdf]

Supplementary Table 1

ID	Age [years]	Gender	Elevated serum IgE	Eczema	Recurrent bacterial skin infections	Recurrent sinusitis/ otitis	Recurrent Pneumonia	Bronchi-ectasis	Muco-cutaneous candidiasis	Other associated skeletal findings	Heterozygous STAT3 mutation
**#1**	37	female	yes	yes	yes	yes	yes	yes	yes	yes	c.1144C>T; p.R382W
**#2**	5	male	yes	yes	yes	yes	no	no	yes	yes	c.1144C>T; p.R382W
**#3**	17	male	yes	yes	yes	yes	yes	yes	yes	yes	c.1145G>A; p.R382Q
**#4**	12	female	yes	yes	yes	yes	yes	yes	yes	yes	c.1909G>A; p.V637M
**#5**	32	male	yes	yes	yes	yes	yes	yes	yes	yes	c.1152T>A; p.F348L
**#6**	16	female	yes	yes	yes	yes	yes	yes	no	no	c.1145G>A; p.R382Q
**#7**	19	female	yes	yes	yes	yes	yes	yes	no	no	c.2114 A>C; p.Y705C
**#8**	23	female	yes	yes	yes	yes	yes	yes	no	yes	c.1406A>G; p.Q469R
**#9**	24	female	yes	yes	yes	yes	yes	no	yes	yes	c.1145G>A; p.R382Q
**#10**	7	male	yes	yes	no	yes	yes	no	no	yes	c.1145G>A; p.R382Q
**#11**	9	male	yes	yes	yes	yes	no	no	yes	yes	c.1145G>A; p.R382Q
**#12**	8	male	yes	yes	yes	yes	yes	no	yes	yes	c.1144C>T; p.R382W
**#13**	48	male	yes	yes	yes	yes	yes	no	yes	yes	c.1144C>T; p.R382W
