# Supplementary material for: Retained primary teeth in STAT3 hyper-IgE syndrome: early intervention in childhood is essential
Source: Orphanet J Rare Dis. 2020 Sep 10;15:244. doi: 10.1186/s13023-020-01516-3 (PMC7488068; doi:10.1186/s13023-020-01516-3)
Supplement: Supplementary file 2 — Additional file 2: Supplementary Table 2. Dental findings in panoramic radiographs. [file 13023_2020_1516_MOESM2_ESM.pdf]

Supplementary Table 2

ID	Years of age at date X-ray taken	Analog/ Digital	Primary tooth in situ	Pathological retained permanent tooth	Physiological retained permanent tooth	Agenesis	Conservative dentistry findings	Orthodontic findings	No resorption of primary tooth root	Resorption of primary tooth root
#3	15	digital	C/B/A<br>H/I/J<br>M/L/K<br>R/S/T	6/5/4/2<br>11/12/13/15<br>22/21/20<br>27/28/29	1<br>16	17<br>32	–	–	C<br>M<br>R	B/A<br>H/I/J<br>L/K<br>S/T
#3	16	analog	A<br>J<br>K<br>T	6/5/4/2<br>11/12/13/15<br>22/21/20<br>27/28/29	1<br>16	17<br>32	–	–	–	A<br>J<br>K<br>T
#4	8	digital	E/D/C/B/A<br>F/G/H/I/J<br>N/M/L/K<br>Q/R/S/T	8/7<br>9/10<br>23<br>26	6/5/2<br>11/12/15<br>22/21/20/18<br>27/28/29/31	4/1<br>13/16<br>17<br>32	–	–	D/C<br>G/H/J<br>M/L/K<br>R/S/T	E/B/A<br>F/I<br>N<br>Q
#4	11	digital	D/C/B/A<br>G/H/I/J<br>M/L/K<br>R/S/T	7<br>10	6/5/2/1<br>11/12/15/16<br>22/21/20/18<br>27/28/29/31	4<br>13<br>17<br>32	–	–	C<br>H/J<br>M/L/K<br>R/S/T	D/B/A<br>G/I
#4	11	digital	C/B/A<br>H/I/J<br>M/L/K<br>R/S/T	–	6/5/2/1<br>11/12/15/16<br>22/21/20/18<br>27/28/29/31	4<br>13<br>17<br>32	–	–	K<br>T	C/B/A<br>H/I/J<br>M/L<br>R/S
#4	12	digital	C/B/A<br>H/I/J<br>M/L/K<br>R/S/T	5<br>12<br>22<br>27	6/2/1<br>11/15/16<br>21/20/18<br>28/29/31	4<br>13<br>17<br>32	–	–	K<br>T	C/B/A<br>H/I/J<br>M/L<br>R/S
#5	30	analog	–	–	–	–	filling<br>3/2/14/22/18<br>/30/31<br>missing<br>13/15/29<br>endodontic<br>treatment<br>20/19<br>bridge 10-12	–	–	–
#6	10	digital	A<br>I/J<br>L/K<br>T	–	5/4/2/1<br>11/12/13/15/16<br>22/21/20/18/17<br>28/29/31/32	none	–	–	K	A<br>I/J<br>L<br>T
#6	12	digital	none	–	2/1<br>16<br>21/17<br>31/32	none	–	–	–	–
#6	15	digital	none	2	1<br>16<br>17<br>32	none	–	–	–	–
#7	12	digital	C/B/A<br>H/I/J<br>M/L/K<br>R/S/T	5<br>12<br>22<br>27	6/4/2/1<br>11/13/15/16<br>21/20/18/17<br>28/29/31/32	shape abnormality 29	–	–	K<br>T	C/B/A<br>H/I/J<br>M/L<br>R/S
#7	14	digital	C/B/A<br>J<br>K<br>T	6/5/4<br>11/12/13<br>20<br>28/29	1<br>15/16<br>18/17<br>31/32	shape abnormality 29	–	–	T	C/B/A<br>J<br>K
#7	19	digital	none	31	1<br>16<br>17<br>32	shape abnormality 29	filling 18	multi-bracket appliance	–	–

#8	15	digital	C	6	1<br>16<br>17<br>32	none	filling 3/ 19	–	–	C
#8	16	digital	none	–	1<br>16<br>17<br>32	none	filling 3/19	multi-bracket appliance	–	–
#8	17	digital	none	–	1<br>16<br>17<br>32	none	filling 3/19	–	–	–
#9	9	digital	D/C/B/A<br>G/H/I/J<br>N/M/L/K<br>Q/R/S/T	7<br>10<br>23<br>26	6/5/2<br>11/12/15<br>22/21/20/18<br>27/28/29/31	none	–	–	D/C<br>G/H/J<br>M/L/K<br>R/S/T	E/B/A<br>F/I<br>N<br>Q
#9	11	digital	C/A<br>H/I/J<br>M/K<br>R/T	–	6/4/2<br>11/12/13/15<br>22/21/20/18<br>27/28/29/31	none	–	–	K<br>R/T	C/A<br>H/J<br>M
#9	13	digital	A<br>J<br>K<br>T	4<br>13<br>20<br>28/29	2/1<br>15/16<br>17<br>32	none	–	–	K<br>T	A<br>J
#9	14	digital	none	4<br>13<br>20	1<br>16<br>17<br>32	none	–	missing 5,12,21,28	–	–
#11	9	digital	D/C/B/A<br>F/G/H/I/J<br>M/L/K<br>R/S/T	9	7/6/5/4/3<br>10/11/12/13/15<br>22/21/20/18<br>27/28/29/31	1<br>16<br>17<br>32	–	–	D/C/B/A<br>G/H/I/J<br>M/L/K<br>R/S/T	F
#12	8	analog	E/D/C/B/A<br>F/G/H/I/J<br>O/N/M/L/K<br>P/Q/R/S/T	24<br>25	8/7/6/5/2<br>9/10/11/12/13/15<br>23/22/21/20/18<br>26/27/28/29/31	4	–	–	D/C<br>G/H<br>N/K<br>Q/R/T	E/B/A<br>F/I/J<br>O/M/L<br>P/S
