# Supplementary material for: Retained primary teeth in STAT3 hyper-IgE syndrome: early intervention in childhood is essential
Source: Orphanet J Rare Dis. 2020 Sep 10;15:244. doi: 10.1186/s13023-020-01516-3 (PMC7488068; doi:10.1186/s13023-020-01516-3)
Supplement: Supplementary file 3 — Additional file 3: Supplementary Table 3. Dental findings in periapical radiographs. [file 13023_2020_1516_MOESM3_ESM.pdf]

Supplementary Table 3

ID	Years of age at date X-ray taken	Analog/Digital	Type of X-ray	Conservative dentistry findings	bone findings
#1	31	digital	periapical regio 30	filling 30	-
#1	31	digital	periapical regio 19	filling 20/19/18	apical radiolucency 18
#1	31	digital	periapical regio 14	filling 13/14/15	-
#1	31	digital	periapical regio 3	filling 4/3	apical radiolucency 4
#1	31	digital	periapical regio 4	filling 6/5/3/2<br>endodontic filling 15	apical radiolucency 4
#7	19	analog	periapical regio 18	caries 18<br>multiband treatment	-
#13	43	analog	periapical regio 30	endodontic filling 29/30	-
#13	43	analog	periapical regio 13	filling 12/14/15<br>caries 13	-
#13	47	digital	periapical regio 13	filling 14<br>pin supply and crown 13	-
